# Noradrenaline Release from Locus Coeruleus Terminals in the Hippocampus Enhances Excitation-Spike Coupling in CA1 Pyramidal Neurons Via β-Adrenoceptors

**DOI:** 10.1093/cercor/bhaa159

**Published:** 2020-07-01

**Authors:** Travis J Bacon, Anthony E Pickering, Jack R Mellor

**Affiliations:** 1 Centre for Synaptic Plasticity, University of Bristol, Bristol, UK; 2 School of Physiology, Pharmacology & Neuroscience, University of Bristol, Bristol BS8 1TD, UK; 3 Bristol Anaesthesia, Pain & Critical Care Sciences, Translational Health Sciences, Bristol Medical School, University of Bristol, Bristol BS2 8HW, UK

**Keywords:** CA1, hippocampus, locus coeruleus, noradrenaline, synaptic transmission

## Abstract

Release of the neuromodulator noradrenaline signals salience during wakefulness, flagging novel or important experiences to reconfigure information processing and memory representations in the hippocampus. Noradrenaline is therefore expected to enhance hippocampal responses to synaptic input; however, noradrenergic agonists have been found to have mixed and sometimes contradictory effects on Schaffer collateral synapses and the resulting CA1 output. Here, we examine the effects of endogenous, optogenetically driven noradrenaline release on synaptic transmission and spike output in mouse hippocampal CA1 pyramidal neurons. We show that endogenous noradrenaline release enhances the probability of CA1 pyramidal neuron spiking without altering feedforward excitatory or inhibitory synaptic inputs in the Schaffer collateral pathway. β-adrenoceptors mediate this enhancement of excitation-spike coupling by reducing the charge required to initiate action potentials, consistent with noradrenergic modulation of voltage-gated potassium channels. Furthermore, we find the likely effective concentration of endogenously released noradrenaline is sub-micromolar. Surprisingly, although comparable concentrations of exogenous noradrenaline cause robust depression of slow afterhyperpolarization currents, endogenous release of noradrenaline does not, indicating that endogenous noradrenaline release is targeted to specific cellular locations. These findings provide a mechanism by which targeted endogenous release of noradrenaline can enhance information transfer in the hippocampus in response to salient events.

## Introduction

The locus coeruleus (LC) is the major source of noradrenergic innervation projecting widely and diffusely throughout the brain (Berridge and Waterhouse 2003; Schwarz et al. 2015; Kebschull et al. 2016). LC neurons respond to novel or salient events, and the release of noradrenaline (NA) is proposed to signal that new schemas need to be formed (Aston-Jones and Cohen 2005; Bouret and Sara 2005; Yu and Dayan 2005; Sales et al. 2019). To achieve this, NA must activate brain areas associated with memory formation and reconfigure information processing to prioritize new information over internal representations. In addition, despite exhibiting very little activity during sleep, LC activity is critical in sleep–wake transitions in response to sensory input (Hayat et al. 2020) and exhibits tonic firing during exploration (Vankov et al. 1995). Therefore, LC activity plays a role in integrating real-time sensory input into cortical representations and decisions.

The hippocampus is important for memory formation, consolidation, and retrieval (Bird and Burgess 2008), and all subfields receive dense noradrenergic innervation (Walling et al. 2012; Schwarz et al. 2015; Wagatsuma et al. 2018). Rodents with disrupted LC input (Compton et al. 1995; Takeuchi et al. 2016), blockade of hippocampal adrenoceptors (Ji et al. 2003; Lemon et al. 2009) or deletion of NA (Murchison et al. 2004) all have impairments in memory and learning, whereas stimulation of LC fibers in the hippocampus reorganizes place cell representations and enhances memory (Takeuchi et al. 2016; Kaufman et al. 2020), leading to the idea that NA is an integral regulator of memory processing. However, the key targets for NA release and the mechanisms by which NA reconfigures neuronal networks in the hippocampus are not well understood.

NA signals via 3 principle G-protein-coupled receptor subtypes—α1-, α2-, and β-adrenoceptors (ARs) (Ramos and Arnsten 2007). β-ARs can be further sub-divided into β1-, β2-, and β3-ARs, which, despite all signaling via the G_s_ protein, have distinct downstream targets (Madison and Nicoll 1982, 1986; Hagena and Manahan-Vaughan 2017; Liu et al. 2017). Within the hippocampus, the major effects of NA on cellular excitability are mediated via β-ARs where the most robust effect is inhibition of the slow afterhyperpolarization (sAHP) (Madison and Nicoll 1982; Haas and Konnerth 1983; Sah and Isaacson 1995), which has been correlated with increased spiking and enhanced learning (Oh et al. 2003; Disterhoft and Oh 2006). In addition, NA also enhances intrinsic excitability via inhibition or downregulation of voltage-gated potassium channels located on the dendrites of CA1 pyramidal neurons (Yuan et al. 2002; Liu et al. 2017). In contrast, activation of α2-ARs hyperpolarizes neurons thereby reducing excitability (Madison and Nicoll 1986; Ul Haq et al. 2012). It is not clear which of these possible mechanisms is engaged by endogenous release of NA.

NA also modulates synaptic communication within hippocampal networks where it facilitates long-term potentiation (LTP) via β-AR enhancement of GluA1 surface expression (Hu et al. 2007; Hagena and Manahan-Vaughan 2012; O'Dell et al. 2015; Hansen and Manahan-Vaughan 2015a; Liu et al. 2017; Palacios-Filardo and Mellor 2019). However, NA has mixed acute effects on basal synaptic transmission. NA has either negligible or small depressant effects on excitatory inputs in the hippocampus (Heginbotham and Dunwiddie 1991; Katsuki et al. 1997), yet despite this can significantly augment population spikes (Dunwiddie et al. 1992; Raman et al. 1996; Lin et al. 2003; O'Dell et al. 2010; Murchison et al. 2011; Ul Haq et al. 2012; Liu et al. 2017). Within inhibitory networks, α1-ARs not only depolarize interneurons and enhance inhibitory drive onto CA1 pyramidal neurons but also depress evoked inhibitory synaptic transmission (Madison and Nicoll 1988; Doze et al. 1991; Bergles et al. 1996). The overall effect of NA on excitatory:inhibitory ratio is unknown, and it is unclear whether acute effects on synaptic transmission contribute to the output from CA1 pyramidal neurons.

Previous studies have primarily utilized pharmacological activation of ARs with supra-micromolar NA concentrations or specific AR agonists. However, estimates suggest that endogenous NA concentrations are sub-micromolar (Harley et al. 1996; Muller et al. 2014; Feng et al. 2019). Therefore, we sought to examine the acute effects of endogenous noradrenaline release from LC terminals on synaptic and cellular properties within the CA1 region of the hippocampus to test whether endogenous NA enhances CA1 output. We found that β-AR activation via NA released from LC terminals enhances the spike output of CA1 pyramidal neurons without altering feedforward excitatory or inhibitory synaptic input. Our data confirm estimates of effective endogenous NA in the sub-micromolar (600 nM) concentration range but found that it did not inhibit sAHP current and is therefore targeted to specific cellular domains. Instead, endogenous NA reduced the charge required to trigger action potentials, which enhanced excitation-spike coupling.

## Methods

All experiments were performed in accordance with UK Home Office legislation after approval by the University of Bristol Animal Welfare and Ethics Review Board.

### Stereotaxic Viral Vector Injections

Male mice aged 28–35 days were anesthetized for surgery with an intraperitoneal (IP) injection of a ketamine (70 mg/kg, Vetalar, Pharmacia, UK) and medetomidine (0.5 mg/kg, Domitor, Pfizer, UK) mixture. After positioning in a stereotaxic frame (Neurostar drill and injection robot), a burr hole (∅ 1.0 mm) was made over the right LC at coordinates from lambda, AP: 4.12 mm (corrected using variation from the average bregma-lambda distance of 4.2 mm, Franklin and Paxinos Mouse Atlas, 3rd edn), ML: 1 mm. A glass micropipette with a tip diameter of 20–30 μm pulled on a vertical puller (Harvard) containing the viral vector was advanced with a 20°-rostral angulation to a depth of 3.9 mm from brain surface. Three injections (250 nL each, at a speed of 125 nL/min) of the CAV2-PRS-ChR2-mCherry viral vector that expresses mCherry tagged ChR2 selectively in noradrenergic neurons (Li et al. 2016) were made at 3.5, 3.7, and 3.9 mm depths.

### Ex Vivo Slice Preparation

Transverse dorsal hippocampal slices were prepared from P21 to P70 C57BL/6J male mice. Following cervical dislocation, brains were immediately removed and immersed in ice-cold sucrose-based cutting solution containing the following (in mM): 205 sucrose, 10 glucose, 26 NaHCO_3_, 2.5 KCl, 1.25 NaH_2_PO_4_, 0.5 CaCl_2_, and 5 MgSO_4_. Individual hippocampi were dissected and mounted on agar and 400-μm slices were cut using a VT1200 vibratome (Leica). Following dissection, slices were transferred to aCSF containing the following (in mM): 119 NaCl, 10 glucose, 26 NaHCO_3_, 2.5 KCl, 1 NaH_2_PO_4_, 1.3 MgSO_4_, and 2.5 CaCl_2_, maintained at ~35 °C for 30 min, and then stored at room temperature. Slices were left for a minimum of 1 h after dissection before recordings were made. All solutions were saturated with 95% O_2_ and 5% CO_2_.

### Electrophysiological Recordings

Slices were submerged in a recording chamber (~3 mL) perfused at 2–4 mL/min with aCSF (as above) at 31–32 °C. CA1 pyramidal cells were visualized using infrared-differential interference contrast (DIC) optics on an Olympus BX-50WI microscope. Patch electrodes with a resistance of 2–8 MΩ were pulled from borosilicate filamented glass capillaries (1.5 OD × 0.86 ID × 100 L mm, Harvard Apparatus) using a vertical puller (PC-10; Narishige) or horizontal puller (P-87, Sutter Instruments). For voltage-clamp synaptic recordings, pipettes were filled with intracellular solution containing the following (mM): 130 CsMeSO_3_, 4 NaCl, 10 HEPES, 0.5 EGTA, 10 TEA, 2 Mg-ATP, 0.5 Na-GTP, 1 QX-314, at pH 7.3, 280–290 mOsm. For voltage-clamp sAHP current recordings pipettes were filled with intracellular solution (mM): 117 KMeSO_3_, 8 NaCl, 10 HEPES, 4 Mg-ATP, 0.3 Na-GTP, 0.2 EGTA, and 1 MgCl, pH 7.4, 280–290 mOsm. For current-clamp recordings, pipettes were filled with intracellular solution (mM): 125 K-gluconate, 5 NaCl, 1 MgCl_2_, 10 HEPES, 0.2 EGTA, 4 Mg-ATP, 0.3 Na-GTP, pH 7.4, 280–290 mOsm. Whole-cell patch-clamp recordings from CA1 pyramidal neurons were made with an AxoPatch 200B amplifier (Molecular Devices), and the current signals were low-pass filtered at 2 or 5 kHz (synaptic currents) or 1 kHz (sAHP currents), and digitized at 10 kHz (synaptic recordings) or 50 kHz (current clamp recordings) using a CED Power 1401 data acquisition board and Signal acquisition software (CED). Junction potentials were not corrected for.

To examine changes in spike probability in response to Schaffer collateral synaptic input, 10 square voltage stimuli (0.1 ms) at 10 Hz were delivered to axons in the stratum radiatum using a bipolar tungsten microelectrode. The stimulation intensity was adjusted such that approximately half of the resulting excitatory post-synaptic potential (EPSP) responses yielded action potentials (i.e., spike probability (*P*_spike_) = 0.5). Following bath application of NA, current injection to the soma was used to adjust the membrane potential (*V*_m_) back to its baseline value to determine whether any effects of NA were mediated by the change in *V*_m_. Throughout experiments, input resistance (*R*_in_) was monitored with hyperpolarizing current injections of 25 pA.

To induce endogenous NA release from LC terminals, hippocampal slices were stimulated with 5-ms duration pulses from a 473-nm LED (2.3 mW/mm^2^, Thor Labs) delivered through the objective lens. Tonic NA release was mimicked by 1 Hz repetitive stimulation for 10 min whereas phasic NA release was induced by 10 light pulses every 20 s at 25 Hz for 10 min. Both protocols finished 10 ms prior to the start of Schaffer collateral stimulation in each recording sweep. In all slices used for endogenous NA release, expression of ChR2-mCherry was confirmed post hoc by immunohistochemistry. Control experiments for light stimulation were conducted in naïve slices from animals that did not receive virus injection.

To measure feedforward excitatory and inhibitory synaptic input, CA1 pyramidal neurons were clamped at the GABA_A_ receptor reversal potential (*E*_GABA_, ~−65 mV, measured separately in experiments using 10 μM NBQX) or the AMPA receptor reversal potential (*E*_AMPA_, ~+5 mV, measured separately in experiments using 50 μM of picrotoxin and 1 μM CGP-55845) to isolate excitatory and inhibitory post-synaptic currents (EPSCs and IPSCs) in response to stimulation of Schaffer collateral axons, respectively (5 pulses at 10 Hz, every 20 s). Stimulation intensity was adjusted to obtain EPSC or IPSC amplitudes of 50–150 pA. Following IPSC recordings, the AMPA/kainate receptor antagonist NBQX (10 μM) was applied to confirm IPSCs were generated by feedforward inhibition resulting from Schaffer collateral stimulation and not direct stimulation of the interneurons. Responses not inhibited by ≥75% within 10 min of NBQX application were discarded from subsequent analysis. Series resistance was monitored throughout voltage-clamp experiments, and synaptic recordings were rejected from analysis if the series resistance increased by more than 30% or greater than 30 MΩ. sAHP currents were elicited by depolarizing pyramidal cells by 70 mV from a holding potential of −50 mV for 120 ms every 20 s. Assessment of excitation-spike coupling was performed by 1-s depolarizing current injections with the current amplitude adjusted to be just supra-threshold for action potential initiation (rheobase).

### Immunohistochemistry

For perfuse fixation, mice were given a terminal anesthetic (Pentobarbital, Euthatal) and then perfused with 4% paraformaldehyde. The cerebrum and brain stem were separated and post-fixed in 4% PFA for >24 h before cryoprotection in 30% sucrose in 0.1 M phosphate buffer (PB) at least 24 h before slice preparation. Brainstem or hippocampi were then mounted on a cryotome (Leica) using Cryomatrix (ThermoFisher Scientific) and 40 μm (pons) and 50 μm (hippocampus) slices cut then stored in 0.1 M PB + 0.02% NaN_3_. Free-floating slices were washed in 0.1 M PB (3×) before transfer to 50% alcohol:dH_2_O for 30 min and washing again in 0.1 M PB (3×). Slices were then incubated overnight in primary antibodies: rabbit anti-mCherry (1:4000, BioVision) and/or sheep anti-DβH (1:1000, Merck Millipore) in 0.1 M PB + 0.3% Triton X-100 containing 5% donkey or goat serum (Sigma-Aldrich). The following day slices were washed in 0.1 M PB (×3) and then incubated for >3 h with appropriate secondary antibodies: goat anti-rabbit Alexa-Fluor 594 (1:1000, Invitrogen) and/or donkey anti-sheep IgG Alexa-Fluor 488 (1:400, Jackson ImmunoResearch) in 0.1 M PB + 0.3% Triton X-100 containing 2% donkey or goat serum. Slices were then transferred to a final solution (0.1 M PB + 1:10 000 DAPI, Invitrogen) before mounting on slides. Slides were imaged using a Leica fluorescent microscope and LASX imaging software (Leica) and fluorescent neurons were manually counted to examine mCherry and DβH expression.

### Data Analysis

Data were analyzed using Signal software (CED) and custom MATLAB scripts. In spike output experiments, spikes were detected on-line if the recorded voltage passed a threshold (0 mV). Spike probability was calculated as the average number of spikes per train of stimuli over a minute period divided by the number of EPSPs in a train (10). Spike latency was determined off-line using a custom MATLAB script that calculated the time between the stimulation artifact or initiation of current injection and the peak of the action potential. Jitter in spike latency was calculated as the standard deviation of spike latencies for the first spike response to stimulation. Spike threshold was calculated from the first derivative of the voltage trace at the point at which d*V*/d*T* exceeded 30 V/s. The charge transfer required to elicit an action potential was calculated as the area under the voltage trace between the start of current injection and the spike threshold. EPSP amplitudes were measured from average traces pooled from consecutive responses within each minute bin and calculated as the peak voltage reached 5–50 ms after stimulation. Only the first EPSP within a train was analyzed and only if no spike was detected on that EPSP. Input resistance was measured as the steady-state voltage deflection in response to current injections at the start of each sweep. *I*_sAHP_ amplitude was measured as the peak current amplitude 100–500 ms after termination of the depolarizing pulse to ensure clear distinction from the medium afterhyperpolarization and compared to the current value 30 ms before the depolarizing pulse. A mean was then taken of the values 10 ms before and 10 ms after this peak amplitude. EPSC and IPSC amplitudes were measured at the detected peak amplitude 5–20 ms after the stimulation artifact and subtracted from the baseline current immediately before the stimulation artifact. To average across experiments for responses that varied substantially in baseline amplitude, responses were normalized to the average during the 10 min prior to NA application. The paired-pulse ratio (PPR) for PSCs was calculated as the second peak divided by the first peak (*P*_2_/*P*_1_).

Experimental unit was defined as cell, and only one cell was recorded per slice. On average ~2 cells were recorded per animal (range 1–4). Data were analyzed blind to the expression of ChR2. Data are plotted as the mean ± standard error of the mean (SEM). Data points on bar graphs used in analysis are the average of 5 min periods from each cell representing the experimental condition. Paired Student’s *t*-tests were used to compare normalized changes from baseline, whereas unpaired Student’s *t*-tests were used when comparing changes between two experimental groups (unless data were not normally distributed, wherein a Mann-Whitney test was used instead). Repeated-measures ANOVA with Tukey’s multiple comparison tests were used to analyze data involving multiple experimental conditions (i.e., baseline, drug/optostimulation, *V*_m_ reset). A one-way ANOVA with a Dunnett’s multiple comparison test was used to compare the different experimental conditions to a WT control group. ^*^, ^**^, ^***^, and ^****^ denote *P* < 0.05, 0.01, 0.001, and 0.0001, respectively. Data were aggregated in Microsoft Excel, and statistical testing was performed using GraphPad 7. Traces and graphs were generated in GraphPad Prism 7 or MATLAB 2017a.

### Drugs and Reagents

DL-noradrenaline hydrochloride and DL-propranolol hydrochloride were purchased from Sigma-Aldrich, fresh stock solutions (50 mM) were made up in dH_2_O each day and stored on ice. Picrotoxin (Sigma-Aldrich), CGP-55845 (HelloBio) and NBQX di-sodium salt (HelloBio) stock solutions (5 mM, 1 mM, and 20 μM, respectively) were made up in dH_2_O and stored at −20 °C.

## Results

### Optoactivation of LC Neurons

**Figure 1:**
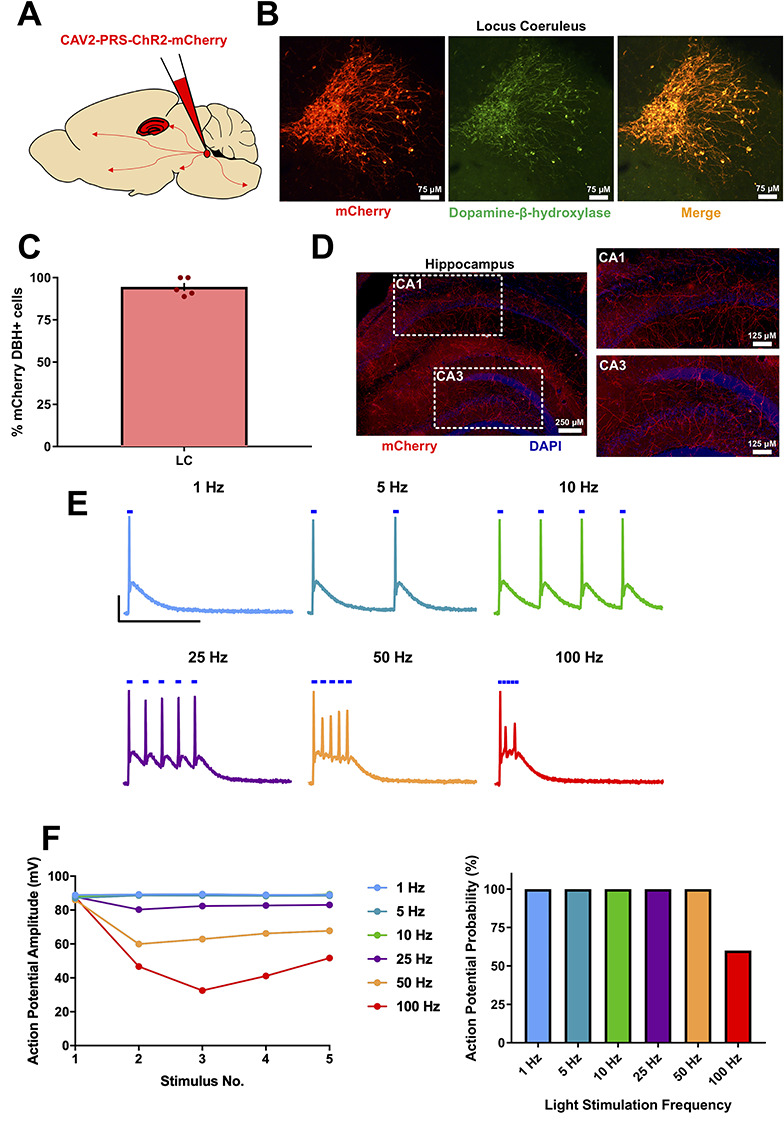
Characterization of the CAV2-PRS-mCherry-ChR2 viral vector expression in mice. (*A*) Schematic for viral vector injections into the Locus Coeruleus (LC). (*B*) mCherry fluorescence co-localized with dopamine-β-hydroxylase fluorescence, confirming successful noradrenergic neuronal targeting of the viral vector. (*C*) % of mCherry^+^ neurons also showing dopamine-β-hydroxylase expression. (*D*) mCherry fluorescence in LC fibers within the hippocampus confirming expression of ChR2 in axons. (*E* and *F*) Light stimulation of ChR2-mCherry-expressing LC neurons recorded in current-clamp configuration reliably elicited action potentials with stimulation frequencies up to 25 Hz after which amplitude and fidelity began to deteriorate. Scale bars = 25 mV, 200 ms.

To measure the effects of endogenous NA release from LC fibers in the hippocampus, channelrhodopsin (ChR2) was expressed in noradrenergic neurons in LC by injection of CAV-PRS-ChR2-mCherry viral vector in mice (Li et al. 2016; Hirschberg et al. 2017) (Fig. 1*A*). 3 weeks after injection, strong ChR2-mCherry expression was found in cell bodies and fibers within the LC that co-localized almost exclusively with dopamine-β-hydroxylase (Fig. 1*B*,*C*; 94.5 ± 2.3% mCherry-expressing cells also stained positive for DβH, *n* = 5). Axonal fibers expressing ChR2-mCherry were also found in a dense network throughout all regions of the hippocampus (Fig. 1*D*). ChR2-mCherry expression was confirmed in all animals and slices used for subsequent electrophysiological experiments measuring the impact of endogenous NA release. Whole-cell current-clamp recordings from ChR2-mCherry-expressing LC neurons showed that brief 5-ms light pulses reliably elicited action potentials up to 25-Hz stimulation frequency but above this frequency action potentials became attenuated in amplitude and fidelity (average for each stimulation across 3 trials shown in Fig. 1*E*,*F*).

### Endogenous NA Enhances CA1 Spike Output Via β-AR Activation

**Figure 2:**
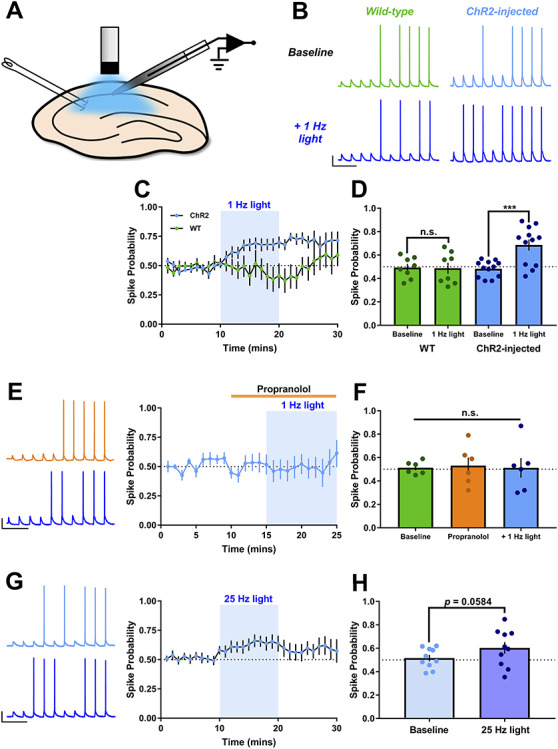
Endogenous NA release enhances CA1 spike output via β-AR activation. (*A*) Schematic showing ex vivo slice recording and optogenetic stimulation. (*B*–*D*) Example traces (*B*), experiment timecourse (*C*) and grouped data (*D*) show 1 Hz tonic light stimulation (10 min, LED) to release endogenous NA enhances the spike probability in CA1 pyramidal neurons in response to Schaffer collateral synaptic stimulation (10 stimuli at 10 Hz) in slices from ChR2-injected but not WT mice. Scale bars = 25 mV, 250 ms. (*E* and *F*) Pre-incubation of slices with the β-AR antagonist, propranolol (500 nM) for 5 min prior to light stimulation blocked the increase in spike probability. Scale bars = 25 mV, 250 ms. (*G* and *H*) Phasic light stimulation (10 pulses at 25 Hz, immediately before synaptic stimuli) also increased spike probability but the increase was more variable. Scale bars = 25 mV, 250 ms.

We next sought to test whether endogenously released NA from LC fibers altered the spike output of CA1 pyramidal neurons in response to synaptic input (Fig. 2*A*). A series of 10 stimuli at 10 Hz were given to the Schaffer collateral fibers with the stimulation intensity adjusted such that approximately five of the 10 elicited EPSPs generated a spike (i.e., spike probability (*P*_spike_) = 0.5). This gave us the opportunity to identify both positive and negative modulation of spike output. Optogenetic stimulation (1 Hz for 10 min) enhanced spike probability of CA1 neurons in slices taken from animals expressing ChR2 in LC by 42.3 ± 8.6% but not in slices from naïve animals and therefore not expressing ChR2 (Fig. 2*B*–*D*; 1 Hz ChR2, *P*_spike_ = 0.69 ± 0.05, *n* = 12, *P* = 0.0003 compared to baseline; 1 Hz non-ChR2, *P*_spike_ = 0.49 ± 0.05, *n* = 8, *P* = 0.835 compared to baseline). This enhancement of spike output by endogenous NA release was completely blocked by application of the β-AR-selective antagonist, propranolol (500 nM) applied 5 min prior and during light stimulation (Fig. 2*E*,*F*; 1 Hz propranolol, *P*_spike_ = 0.51 ± 0.08, *n* = 6, *P* = 0.903 compared to baseline). This indicates that the enhancement of spike output is mediated by activation of β-ARs and also that there is little endogenous NA tone within the slices.

LC noradrenergic neurons exhibit two distinct firing modes often classed as tonic and phasic equating to prolonged firing at ~1 Hz (tonic) or burst firing at ~25 Hz (phasic) (Berridge and Waterhouse 2003; Aston-Jones and Cohen 2005; Bouret and Sara 2005; Vazey et al. 2018). These different modes will likely lead to different NA concentration profiles, which may change the effect of NA in a similar manner to that seen for dopamine release in the hippocampus (Rosen et al. 2015). We therefore next mimicked phasic release of NA using bursts of 10 light pulses at 25 Hz repeated every 20 s in slices from ChR2 expressing animals. Phasic NA release caused a similar but slightly smaller enhancement of spike output (Fig. 2*G*,*H*; *P*_spike_ = 0.59 ± 0.048, *n* = 11, *P* = 0.058 compared to baseline) compared to tonic (1 Hz) release. We therefore used tonic 1 Hz stimulation as the more effective stimulation frequency for subsequent experiments testing the effects of endogenous NA release.

### Dose-Dependent Enhancement of CA1 Spike Output by Exogenous NA

The presence of ARs, potentially at some distance from NA terminals and release sites, makes it difficult to determine the precise effective concentration of endogenous NA at receptors. Reports suggest that NA release from LC terminals is likely in the sub-micromolar to low micromolar range (Harley et al. 1996; Muller et al. 2014; Feng et al. 2019) but this may vary depending on the brain region, the neuron type, and the target receptors. To estimate the concentration of endogenous NA release in our experiments, we explored the dose-response relationship for exogenous bath-applied NA concentrations ranging from 200 nM to 20 μM assayed on CA1 spike output.

**Figure 3:**
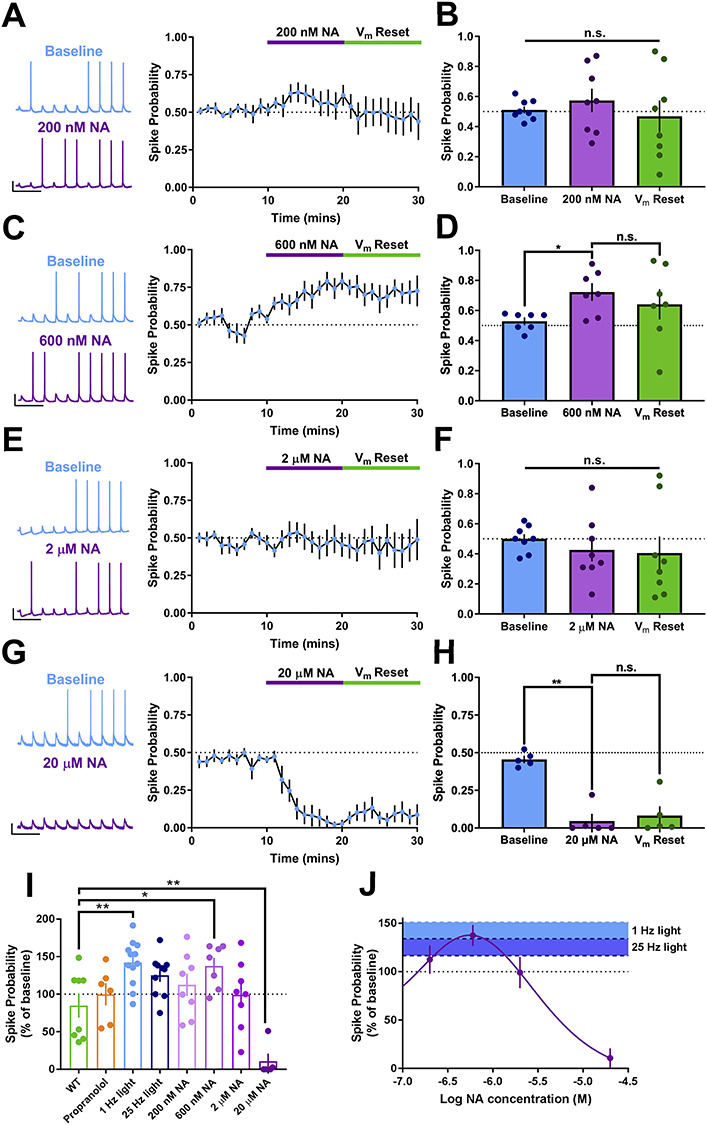
Biphasic dose-response relationship for exogenous NA on CA1 spike output. (*A*–*H*) Example traces, averaged timecourse and grouped data for spike probability experiments using exogenous application of 200 nM (*A* and *B*), 600 nM (*C* and *D*), 2 μM (*E* and *F*) and 20 μM NA (*G* and *H*). (*I*) Percentage change in spike probability for all conditions. (*J*) Dose-response curve for change in spike probability with concentration of NA. Overlaid blue shading indicates the mean and SEM change in spike probability produced by 1 or 25 Hz light stimulation of endogenous NA release. Overlap between blue shading and dose-response curve indicates likely concentration of endogenous NA release. Scale bars = 20 mV, 250 ms.

200 nM NA had no effect on spike probability (Fig. 3*A*,*B*; *P*_spike_ = 0.57 ± 0.08, *n* = 8, *P* = 0.702 compared to baseline). However, 600 nM NA produced a robust increase in the spike output of CA1 pyramidal neurons by 37.6 ± 10.9% (Fig. 3*C*,*D*; *P*_spike_ = 0.72 ± 0.05, *n* = 7, *P* = 0.029 compared to baseline). This increase was accompanied by a small and variable depolarization of the resting membrane potential (Δ*V*_m_ = +2.85 ± 1.55 mV, *P* = 0.114). In recordings where neurons depolarized, *V*_m_ was reset after 10 min to test whether the enhanced spike probability was due to depolarization but resetting *V*_m_ did not reverse the increase in spike probability (*P*_spike_ = 0.64 ± 0.10, *n* = 7, *P* = 0.481 compared to 600 nM NA). Surprisingly, when the concentration of exogenous NA was increased to 2 μM the enhancement of spike probability disappeared and this was not affected by resetting *V*_m_ (Fig. 3*E*,*F*; *P*_spike_ = 0.43 ± 0.08, *n* = 8, *P* = 0.934 compared to 2 μM NA). Furthermore, when the concentration of exogenous NA was further increased to 20 μM spike probability was dramatically reduced to 10.7 ± 10.1% (Fig. 3*G*,*H*; *P*_spike_ = 0.05 ± 0.04, *P* = 0.003 compared to baseline, *n* = 5). The reduction in spike probability was accompanied by a variable hyperpolarization of CA1 pyramidal neurons (Δ*V*_m_ = −2.06 ± 1.31 mV, *n* = 5, *P* = 0.187 compared to baseline), but resetting *V*_m_ did not reverse the reduction in spike probability (0.08 ± 0.06, *P* = 0.877 compared to 20 μM NA).

To compare changes in spike probability and construct a dose-response relationship we normalized changes in spike probability for each cell and averaged for each condition (Fig. 3I). This revealed a biphasic dose-response relationship for exogenous NA (Fig. 3J), from which it was possible to estimate the effective concentration of endogenous optogenetically-evoked NA. Endogenous NA evoked with 1 Hz optostimulation (light blue bar is the ± SEM range of this result set) yielded a change in spike probability comparable to the peak of the biphasic dose-response curve corresponding to 600 nM exogenous NA, providing a best estimate for the concentration of endogenous NA.

### Effects of NA on Feedforward Excitatory and Inhibitory Synaptic Inputs to CA1

An enhancement of spike probability and excitation-spike coupling could result from changes in synaptic input to CA1 neurons or from changes to CA1 neuronal intrinsic excitability and synaptic integration. We assessed which of these factors is the key mediator of enhanced spike probability, focusing first on synaptic inputs.

**Figure 4:**
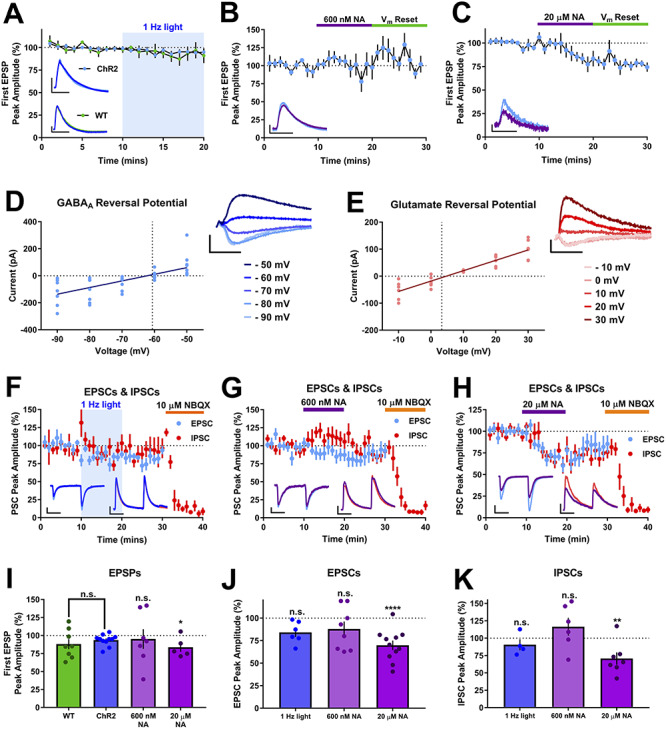
Endogenous NA release has no effect on Schaffer collateral feedforward excitatory and inhibitory synaptic transmission. (*A*–*C*) EPSPs recorded during spike probability experiments were unaffected by endogenous NA release (*A*) or 600 nM NA (*B*) but depressed by 20 μM NA (*C*). Plots in A show EPSPs recorded in slices from virus injected and WT animals. Insets in panels show example EPSPs. Scale bars = 2 mV, 20 ms. (*D* and *E*) Experimentally determined reversal potentials for GABA and glutamate receptors, respectively. Current-voltage plots and example traces for IPSCs (*D*) and EPSCs (*E*). Scale bars = 200 pA and 100 ms (GABA_A_ reversal traces), 50 pA and 100 ms (glutamate reversal traces). (*F*–*H*) Time-course plots showing EPSC and IPSC amplitudes and the effect of endogenous NA release (*F*), 600 nM (*G*) and 20 μM NA (*H*). Scale bars = 100 pA, 50 ms (both traces in H and IPSCs in *F* and *G*), 25 pA, 50 ms (EPSCs, *F* and *G*). (*I*–*K*) Grouped data for EPSPs (*I*), EPSCs (*J*) and IPSCs (*K*) for each NA application.

Within the spike probability experiments it was possible to extract and quantify the strength of the synaptic input by measuring the amplitude of the first EPSP in the stimulus train, since the first EPSP rarely generated an action potential. Analysis of these data revealed that EPSP amplitude was not altered by endogenous NA release or by exogenous NA applied at concentrations of 200 nM, 600 nM or 2 μM, but was reduced by 20 μM NA (Fig. 4*A*–*C*,*I*; endogenous NA = 93.6 ± 2.3% of baseline, *n* = 11, *P* = 0.408 compared to WT; 600 nM NA = 95.13 ± 13.7% of baseline, *n* = 7, *P* = 0.750 compared to baseline; 20 μM NA, 77.16 ± 3.79% of baseline, *n* = 5, *P* = 0.049 compared to baseline), and this EPSP depression was not rescued by resetting *V*_m_. These data suggest one cause of the reduced spike output in response to 20 μM NA is an attenuation of excitatory synaptic input. The data also indicate that enhancement of spike probability by endogenous NA release or 600 nM exogenous NA does not result from increases in excitatory synaptic input.

Using the first EPSP from the spike probability experiments is a useful measure of Schaffer collateral excitatory synaptic input to the CA1, but does not provide information on inhibitory synaptic strength or the overall excitation:inhibition ratio, which may be modulated by NA (Madison and Nicoll 1988; Doze et al. 1991; Bergles et al. 1996). We examined this local microcircuit in more detail using whole-cell voltage-clamp recordings to isolate feedforward excitatory and inhibitory synaptic currents by holding CA1 pyramidal neurons at the experimentally determined reversal potentials for AMPA (~+5 mV) and GABA_A_ (~−65 mV) receptors (Fig. 4*D*,*E*). Inhibitory inputs were confirmed as feedforward disynaptic inputs by application of the AMPA/Kainate receptor antagonist NBQX (10 μM) at the end of each experiment and resulting blockade of IPSCs. Neither endogenous NA release by 1 Hz light stimulation nor 600 nM exogenous NA produced any effect on feedforward excitatory or inhibitory synaptic responses (Fig. 4*F*,*G*,*J*,*K*; 1 Hz light stimulation, EPSCs = 84 ± 5% and IPSCs = 86 ± 8% of baseline, *n* = 6 and 5, respectively, *P* = 0.116 and *P* = 0.310, respectively; 600 nM NA, EPSCs = 89 ± 8% and IPSCs = 110.9 ± 10.6% of baseline, *n* = 8 and 6, respectively, *P* = 0.460 and *P* = 0.231, respectively). However, 20 μM exogenous NA depressed both feedforward EPSCs and IPSCs (Fig. 4*H*,*J*,*K*; EPSCs = 69.8 ± 5.3% and IPSCs = 70.7 ± 8.9% of baseline, *n* = 11 and 7, respectively, *P* = 0.00009 and 0.009, respectively). The reduction in EPSC amplitude with 20 μM NA is comparable to the depression of EPSPs in spike probability experiments. Paired-pulse ratio for EPSCs or IPSCs was not affected by either endogenous or 600 nM or 20 μM exogenous NA (light stimulation = 1.50 ± 0.12 versus baseline = 1.45 ± 0.15, *n* = 6, *P* = 0.557 compared to baseline; 600 nM NA = 1.46 ± 0.12 versus baseline = 1.50 ± 0.01, *n* = 8, *P* = 0.768 compared to baseline; 20 μM NA = 1.48 ± 0.11 versus baseline = 1.48 ± 0.06, *n* = 11, *P* = 0.970 compared to baseline). These data confirm the reduced excitatory synaptic input caused by 20 μM NA and indicate this is a contributor to the reduction in spike output. However, the data also show that endogenous NA release or exogenous NA concentrations that mimic those found endogenously have no acute effect on either excitatory or inhibitory feedforward synaptic transmission. This rules out a role for synaptic input changes underlying the enhancement of spike probability by NA and therefore suggests that changes to intrinsic cellular excitability are the most likely mediators.

### Enhancement of Intrinsic Cellular Excitability by Endogenous NA Release

**Figure 5:**
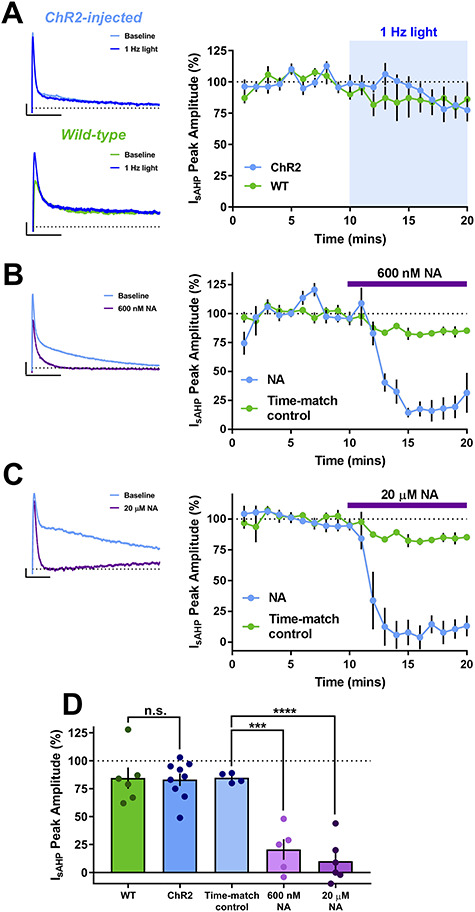
*I*_sAHP_ currents are inhibited by exogenous 20 μM or 600 nM NA, but not endogenous NA release. (*A*–*C*) Timecourse and example traces showing *I*_sAHP_ inhibition with 600 nM NA (*B*) and 20 μM NA (*C*) but not endogenous NA release in slices from virus injected mice or by light stimulation in slices from WT mice (*A*). Scale bars = 10 pA, 500 ms (WT), 25 pA, 500 ms (ChR2-injected), 50 pA, 500 ms (600 nM NA), 10 pA, 250 ms (20 μM NA). (*D*) Grouped data for virus injected and WT mice show no effect of endogenous NA release whereas both 600 nM and 20 μM NA depressed *I*_sAHP_ when compared to a time-matched control.

The best characterized target for β-ARs is inhibition of the sAHP, a Ca^2+^-activated K^+^ current with a slow decay and an important regulator of neuronal excitability (Oh et al. 2003; Disterhoft and Oh 2006). The sAHP can be triggered by repetitive trains of synaptic input or a strong depolarizing current delivered directly to the soma and sAHP inhibition produces a reliable increase in spike output (Madison and Nicoll 1982; Haas and Konnerth 1983; Sah and Isaacson 1995), making it a good candidate to mediate the observed enhancement of spike probability. sAHP currents (*I*_sAHP_) with amplitudes of 10–40 pA were induced with a depolarizing voltage step from −50 to +20 mV. These sAHP currents reduced gradually in amplitude to 84.8 ± 2.2% over 20 min in time matched controls. We next tested the most effective optostimulation protocol for NA release (1 Hz) to determine if endogenous NA could inhibit the sAHP. Surprisingly, NA release elicited by 1 Hz light stimulation had no effect on *I*_sAHP_ amplitude when compared to light stimulation in non-ChR2 expressing controls (Fig. 5*A*,*D*; WT = 83 ± 6% of baseline, ChR2 = 85 ± 10% of baseline, *n* = 6 and 9, respectively, *P* = 0.859). In contrast, exogenous application of 600 nM or 20 μM NA rapidly and substantially inhibited *I*_sAHP_ in comparison to time matched controls (Fig. 5*B*–*D*; 600 nM, 21 ± 9% of baseline, *n* = 5, *P* = 0.0003 compared to time matched control; 20 μM, 10 ± 8% of baseline, *n* = 6, *P* = 0.00009 compared to time matched control). These data indicate that the release of endogenous NA is not sufficient to activate the β-ARs linked with Ca^2+^-activated K^+^ channels underlying the sAHP and that inhibition of sAHP current does not underlie the enhancement of spike probability caused by endogenous NA.

**Figure 6:**
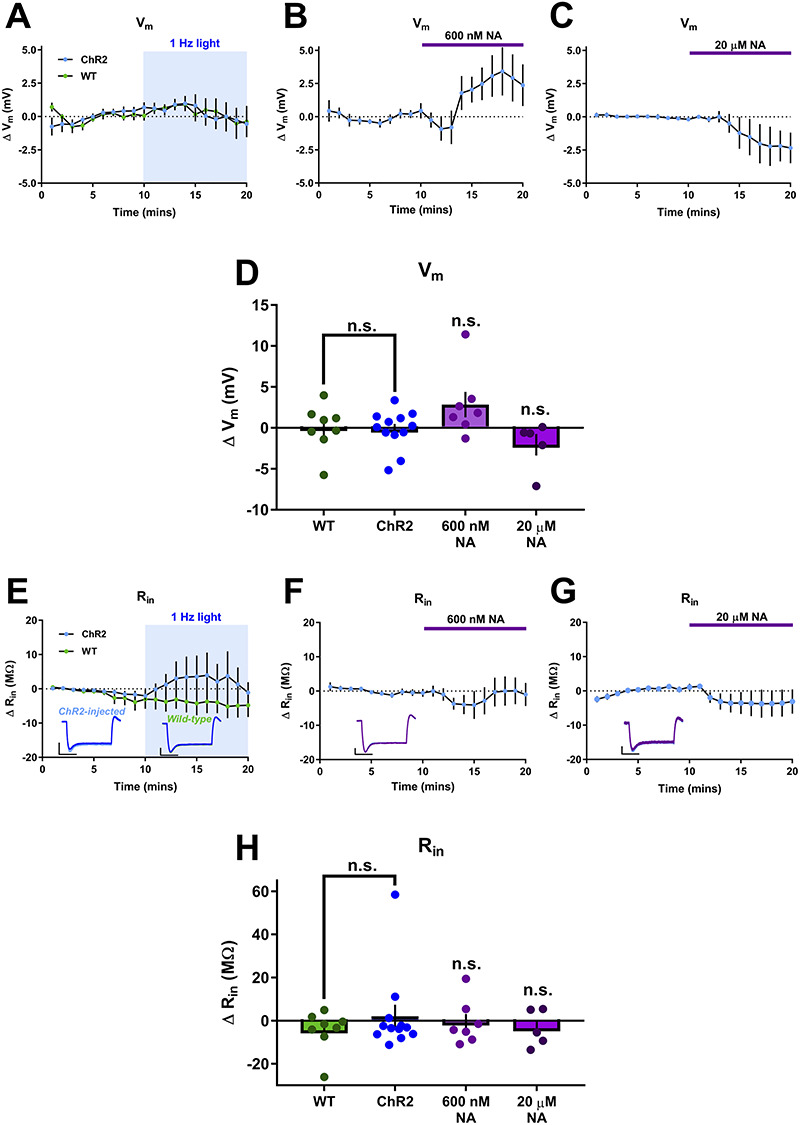
Endogenous NA release does not modulate resting intrinsic cellular properties. (*A*–*C*) Timecourse of changes in resting membrane potential (*V*_m_) from baseline showing no effect of endogenous NA release (*A*) or 600 nM NA (*B*) but a hyperpolarization caused by 20 μM NA (*C*). (*D*) Group data for effects of all conditions on *V*_m_. (*E*–*G*) Timecourse of changes in input resistance (*R*_in_) from baseline showing no effect of endogenous NA release (*E*), 600 nM NA (*F*) and 20 μM NA (*G*). Example traces are inset. Scales bars = 5 mV, 200 ms. (*H*) Group data for effects of all conditions on *R*_in_.

Alternative mechanisms that could cause enhancement of spike probability include modulation of conductances active at rest or those active in response to depolarizing input. To monitor conductances active at rest we analyzed membrane potential and input resistance during the spike probability experiments. Average resting membrane potential was −65.1 ± 0.7 mV and input resistance 90.5 ± 2.6 MΩ and neither endogenous NA release or exogenous application of 600 nM or 20 μM NA produced consistent changes in membrane potential (Fig. 6*A*–*D*; ChR2 = −0.19 ± 0.69 mV, *n* = 12, *P* = 0.891 compared to baseline; WT = −0.01 ± 1.00 mV, *n* = 8, *P* = 0.897 compared to baseline; 600 nM NA = 2.85 ± 1.55 mV, *n* = 7, *P* = 0.114 compared to baseline; 20 μM NA = −2.06 ± 1.31, *n* = 5, *P* = 0.187 compared to baseline) or input resistance (Fig. 6*E*–*H*; ChR2 = 2.01 ± 5.38 MΩ, *n* = 12, *P* = 0.413 compared to baseline; WT = −4.49 ± 3.35 MΩ, *n* = 8, *P* = 0.383 compared to baseline; 600 nM NA = −0.80 ± 3.91, *n* = 7, *P* = 0.688 compared to baseline; 20 μM NA = −3.53 ± 3.82, *n* = 5, *P* = 0.278 compared to baseline) indicating no effect of NA on resting excitability.

**Figure 7:**
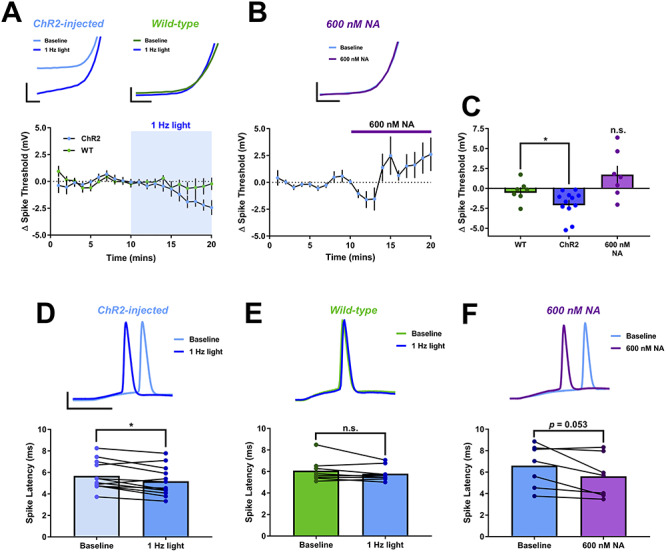
Endogenous NA release reduces spike latency. (*A* and *B*) Spike threshold in response to evoked EPSPs was lowered by endogenous NA release (*A*) but not 600 nM NA (*B*). Spike threshold is reduced in slices from virus injected animals but not in WT animals. Example traces are inset. Scale bars = 5 mV, 0.5 ms for expanded traces and 25 mV, 5 ms otherwise. (*C*) Group data for spike threshold. (*D*–*F*) Group data and example traces showing that spike latency in response to evoked EPSPs is reduced by endogenous NA release in slices from virus injected mice (*D*) or 600 nM NA (*F*) but not by light stimulation in slices from WT mice (*E*). Scale bars = 25 mV, 5 ms.

A final possible mechanism for the enhancement of spike probability by NA is a strengthening of the coupling between excitatory input and action potential generation. Therefore, we analyzed action potential initiation measures including the spike threshold and the spike latency, which is a measure of the amount of charge required to initiate an action potential. The action potential threshold was measured for the first spike in each train of synaptic stimuli before and during release of endogenous NA or application of exogenous 600 nM NA but not 20 μM NA since spiking was almost entirely prevented by application of this dose (Fig. 2). Interestingly, endogenous NA release, but not 600 nM exogenous NA, lowered the threshold for action potential initiation (Fig. 7*A*–*C*; endogenous NA = −1.92 ± 0.47 mV, *n* = 12, *P* = 0.028 compared to wild-type (WT); 600 nM NA = +1.73 ± 1.11 mV, *n* = 7, *P* = 0.155 compared to baseline). Furthermore, spike latency was reduced by both endogenous NA release and exogenous 600 nM NA (Fig. 7*D*–*F*; endogenous NA = 5.68 ± 0.39 to 5.18 ± 0.40, *n* = 12, *P* = 0.0095; 600 nM NA = 6.60 ± 0.75 to 5.62 ± 0.74, *n* = 7, *P* = 0.053 compared to baseline). Taken together, these data indicate a potential modulation of active conductances by NA enhancing the coupling between excitatory input and action potential initiation.

To confirm these findings, we assessed excitation-spike coupling using somatic current injections to initiate action potentials with current injection amplitude defined by the rheobase (the minimum somatic current injection to evoke a single action potential). In CA1 pyramidal neurons, K^+^ channels (predominantly the Kv1 family) expressed in the somatodendritic region are activated near the action potential threshold and cause a delay in action potential spiking, resulting in a longer action potential latency and increased jitter or variance in latency.

**Figure 8:**
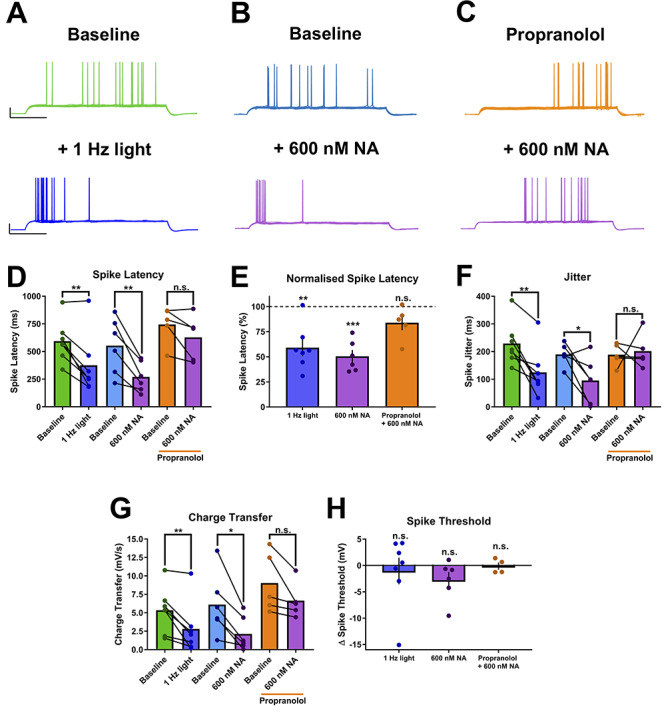
Excitation-spike coupling is enhanced by endogenous NA release. (*A*–*C*) Example traces showing a reduction in rheobase spike latency and jitter in response to endogenous NA release (*A*) or 600 nM NA (*B*) that is blocked with propranolol (*C*). Scale bars = 25 mV, 250 ms. (*D*–*H*) Group data showing the effects of endogenous NA release and 600 nM NA and the block with propranolol on spike latency (*D* and *E*), jitter (*F*), charge transfer required to elicit an action potential (*G*) and spike threshold (*H*).

Similar to action potentials initiated by synaptic stimulation, the latency of action potentials initiated by somatic current injection was reduced by endogenous NA release (Fig. 8*A*,*D*,*E*; 594 ± 71 to 376 ± 104 ms, *n* = 7, *P* = 0.031 compared to baseline), recapitulated by 600 nM exogenous NA (Fig. 8*B*,*D*,*E*; 553 ± 104 to 271 ± 55 ms, *n* = 6, *P* = 0.006 compared to baseline) and blocked by the β-AR antagonist propranolol (500 nM) (Fig. 8*C*,*D*,*E*; 744 ± 75 to 628 ± 93 ms, *n* = 5, *P* = 0.106 compared to baseline). These effects on spike latency were mirrored by the spike jitter (Fig. 8*A*–*C*,*F*; endogenous NA, 229 ± 30 to 125 ± 33 ms, *P* = 0.004 compared to baseline; 600 nM NA, 190 ± 16 to 95 ± 33 ms, *P* = 0.033 compared to baseline; propranolol +600 nM NA, 189 ± 18 to 202 ± 28, *P* = 0.772 compared to baseline). The reduction in spike latency caused by NA could be due to a reduction in action potential threshold or a reduction in the amount of current injection required to reach threshold. In these experiments, the action potential threshold was not altered by NA (Fig. 8*H*; endogenous NA: −1.0 ± 2.5 mV, *P* = 0.697; 600 nM NA = −2.8 ± 1.5 mV, *P* = 0.131; propranolol +600 nM NA = −0.11 ± 0.7 mV, *P* = 0.885) but the amount of charge transfer was reduced (Fig. 8*G*; endogenous NA: 5.3 ± 1.2 to 2.8 ± 1.3 mV/S, *P* = 0.016; 600 nM NA = 6.1 ± 1.7 to 2.1 ± 1.0 mV/S, *P* = 0.008; propranolol +600 nM NA = 9.0 ± 1.8 to 6.64 ± 1.1 mV/S, *P* = 0.087). These results are entirely consistent with NA causing a down-regulation of voltage-dependent K^+^ channels, which reduces the amount of charge required to bring the membrane potential to the action potential threshold (Yuan et al. 2002; Liu et al. 2017).

## Discussion

We demonstrate that endogenous NA release, elicited by optogenetic stimulation of noradrenergic fibers in the hippocampus, enhances CA1 pyramidal neuron spike output in response to Schaffer collateral synaptic input. Like many of the excitatory actions of NA, this effect is mediated via β-ARs (Dunwiddie et al. 1992; Raman et al. 1996; Lin et al. 2003; O'Dell et al. 2010; Murchison et al. 2011; Ul Haq et al. 2012; Liu et al. 2017). The enhancement of spike output was mimicked by application of relatively low concentrations of exogenous NA (600 nM) and but not by higher concentrations (2–20 μM) suggesting that the effective concentration of endogenously released NA is around 600 nM. This agrees very closely with recent estimates of NA release from LC terminals using genetically-encoded sensors (Feng et al. 2019). Surprisingly, this profile of endogenous NA release did not inhibit sAHP currents, even though exogenous NA consistently produces inhibition (Madison and Nicoll 1982; Haas and Konnerth 1983; Sah and Isaacson 1995) and neither did NA have an effect on synaptic input to CA1 pyramidal neurons. Instead the increased spike output resulted from enhancement of excitation-spike coupling consistent with a down-regulation of voltage-dependent K^+^ channels (Liu et al. 2017).

The use of optogenetic techniques to selectively stimulate neuromodulator release has highlighted differences between exogenous bath-applied and endogenous release of dopamine and serotonin in *ex vivo* brain slice experiments (Rosen et al. 2015; Teixeira et al. 2018). We also show that this is the case for NA and propose that such distinct effects are not only concentration-dependent, but also depend on the spatio-temporal release dynamics of neuromodulators. Complete inhibition of the sAHP with saturating concentrations (≥10 μM) of NA have been reported previously (Madison and Nicoll 1982; Haas and Konnerth 1983; Dunwiddie et al. 1992; Sah and Isaacson 1995), and we show much lower concentrations (600 nM) also inhibit *I*_sAHP_. However, the lack of *I*_sAHP_ modulation by endogenously released NA suggests that NA is not released near the β-ARs, which modulate *I*_sAHP_. The precise location of noradrenergic terminals and NA release in CA1 is unknown, but the LC is conventionally thought to modulate this network via bulk “volume transmission” (Fuxe et al. 2010). In contrast, some evidence suggests that CA3 pyramidal neurons are synaptically targeted by the LC for more precise regulation (Walling et al. 2012). However, the dichotomy between exogenous and endogenous NA actions on *I*_sAHP_ suggests there is some degree of compartmental targeting by LC terminals in CA1. This highlights the importance of NA transporters in shaping the spatiotemporal profile of NA by removing NA from the extracellular space. Indeed, application of uptake inhibitors increases the effective concentration (i.e., the concentration experienced by ARs) of endogenous NA released by optogenetic stimulation of LC terminals (Feng et al. 2019). NA transporters are less likely to alter the effective concentration of bath applied exogenous NA since the reserve pool of NA is much larger but this may potentially lead to an overestimation of the effective concentration of bath applied NA. It is also reported that LC terminals in the hippocampus may release dopamine as well as NA and indeed that dopamine mediates the majority of the effects of LC activation (Kempadoo et al. 2016; Takeuchi et al. 2016; Wagatsuma et al. 2018). We find no evidence for dopamine release from LC terminals since all of the effects documented here were blocked by the β-adrenergic antagonist propranolol.

In acute and cultured hippocampal CA1 neurons, Kv1.1, Kv1.2 and Kv1.6 channels are expressed in the somatodendritic and axonal compartments (Grosse et al. 2000), where they regulate spike generation at the axon initial segment (Guan et al. 2007; Liu et al. 2017). Such expression overlaps with that observed for β-ARs (Guo and Li 2007), so allowing rapid modulation of action potential initiation. Since the molecular identity (and therefore subcellular location) of the K^+^ channels underlying the sAHP have yet to be unequivocally defined (Andrade et al. 2012; King et al. 2015; Higham et al. 2019) it is plausible that these channels are located in the more distal dendrites only accessible to exogenous NA acting on β-ARs. In support of this, the sAHP in CA1 pyramidal neurons can be activated by subthreshold EPSPs, suggesting a dendritic location distant from the soma (Lancaster et al. 2001) and estimated to be 100–200 μm from the soma in CA1 pyramidal and rat basolateral amygdala neurons (Sah and Bekkers 1996; Power et al. 2011). Moreover, sAHP inhibition with exogenous NA concentrations as low as 300 nM has been demonstrated (Madison and Nicoll 1982, 1986). These studies support the notion that the present results are not due to optogenetically triggered NA being released at a concentration insufficient to modulate the sAHP, but rather that release is targeted to specific domains of the CA1 cells. Indeed a more distal location is likely given that the sAHP acts not only to regulate neuronal excitability by counteracting repetitive synaptic barrages in the dendrites (Andrade et al. 2012), but also shapes the temporal summation of EPSPs (Lancaster et al. 2001). In addition, the sAHP is inhibited by β1-ARs (Madison and Nicoll 1982, 1986) whereas Kv1.1 channels are inhibited by β2-ARs (Liu et al. 2017) and therefore the location and affinity of β1 and β2 ARs for endogenous NA may also contribute to the differential effect of endogenous NA on sAHP currents and Kv channels. It is also possible that the temporal profile of NA release resulting from tonic versus phasic LC activity could differentially activate receptor populations.

The absence of noradrenergic modulation of *I*_sAHP_ or conductances active at resting membrane potentials indicates that the modulation of spike output is via regulation of excitation-spike coupling, which was supported by the finding that noradrenaline reduces the charge required to drive CA1 pyramidal neurons to fire action potentials. We found this was not due to a consistent reduction in action potential threshold and therefore that noradrenaline is likely modulating voltage-dependent conductances activated by subthreshold depolarization that normally regulate the coupling between sub-threshold excitation (either by synaptic input or somatic current injection) and action potential initiation. Since noradrenaline inhibits dendritic voltage-dependent potassium conductances including Kv4.2 and Kv1.1 (Hoffman and Johnston 1999; Yuan et al. 2002; Liu et al. 2017) that control the coupling between sub-threshold depolarization and action potential initiation (Hoffman et al. 1997) we propose that this is the mechanism by which endogenous NA release modulates excitation-spike coupling but our data do not distinguish which of these two potassium conductances mediate the effects of NA.

Endogenous NA release did not modulate synaptic inputs to CA1 in our experiments and only at higher exogenous concentrations of NA was synaptic transmission depressed. This broadly agrees with previous reports where the effect of NA on synaptic transmission is weak but generally depressing at higher doses (Madison and Nicoll 1988; Doze et al. 1991; Heginbotham and Dunwiddie 1991; Bergles et al. 1996; Katsuki et al. 1997). Interestingly, presynaptic α2-ARs can depress synaptic transmission in LC (Starke and Montel 1973) and are expressed on the terminals of CA3 axons (Milner et al. 1998). However, we found no evidence for a presynaptic depression of synaptic transmission since we saw no change in paired-pulse ratio on addition of 20 μM NA. Therefore, the major role for endogenous NA release on synaptic transmission is likely to be via regulation of long-term synaptic plasticity. Induction of LTP can be facilitated by enhancing postsynaptic excitability via downregulation of Kv1.1 (Liu et al. 2017) or inhibition of sAHP (Fuenzalida et al. 2007), which enhance back-propagating action potentials and widen the temporal window for associative plasticity (Lin et al. 2003; Liu et al. 2017). In addition, induction of LTP may also be facilitated by NA by enhancing Ca^2+^ influx through NMDARs via PKA-mediated phosphorylation (Raman et al. 1996) and expression of LTP can be facilitated by increased trafficking of AMPARs to the postsynaptic membrane as a result of AMPAR phosphorylation (Hu et al. 2007; Makino et al. 2011), DNA methylation and post-translational histone modifications (Maity et al. 2015). Coincident timing of LC activity and Schaffer collateral input drives the induction and regulates the persistency of NMDA receptor-dependent LTP in the CA1 (Lemon et al. 2009; Hansen and Manahan-Vaughan 2015b; Liu et al. 2017). Such intrinsic synaptic activity is absent in the present study, but this would not prevent the modulation of the Kv channels and altered spike probability we observed. Indeed, it is plausible that the coincident timing of LC and SC inputs may augment the effects of one another and lead to a greater degree of spike probability enhancement and downstream plasticity.

The LC typically shows spontaneous tonic spiking activity during wakefulness in the range 0.1–5 Hz (Aston-Jones and Bloom 1981; Vankov et al. 1995; Takeuchi et al. 2016; Xiang et al. 2019). During sleep states, particularly non-REM sleep, the LC is essentially silent, however an increase in LC activity precedes and gates arousal from sleep (Aston-Jones and Bloom 1981; Carter et al. 2010) and a slightly higher tonic level of firing (1–2 Hz) is commonly observed during exploratory behavior (Vankov et al. 1995; Takeuchi et al. 2016). Further, the selective optogenetic addition of a low level of noradrenergic activity during sleep alters hippocampal memory consolidation (Swift et al. 2018). Where phasic burst firing is thought to signal saliency, possibly via a network reset mechanism to allow the learning of a new rule or contingency that modifies place cell representations (Bouret and Sara 2005; Kaufman et al. 2020), within this experiment we found an increase in excitation-spike coupling with a modest stimulation of LC terminals (1 Hz) corresponding to a low level of NA (~600 nM). However, the level of NA in the hippocampal slice under basal conditions was biologically negligible as the application of propranolol was without effect on the excitation-spike coupling in the absence of optogenetic drive. The low frequency optogenetic activation of LC terminals may therefore promote memory consolidation (i.e., of a given environment) into an existing schema without resetting the network. In this context the β-AR mechanism acts to fundamentally alter the input-output relationship of the CA1 gateway for hippocampal information flow to the cortex without altering the synaptic communication within the local microcircuits. As such this represents a novel noradrenergic gating mechanism that can globally boost the output from the hippocampus to the cortex dependent on behavioral context driven by neuromodulator state.

## Author Contributions

T.J.B. designed and performed research, analyzed data, and wrote the paper. A.E.P. and J.R.M. designed and supervised research and wrote the paper.

## Funding

Biotechnology and Biological Sciences Research Council (BBSRC BB/M009122/1); Wellcome Trust (AEP-gr088373, JRM-101029).

## Notes

We thank all members of the Mellor and Pickering groups for constructive discussion and Eric J. Kremer for provision of CAV vectors. *Conflict of Interest*: The authors declare no competing financial interests.
